# Supplementary material for: Characterising school-age health and function in rural Zimbabwe using the SAHARAN toolbox
Source: PLoS One. 2023 May 11;18(5):e0285570. doi: 10.1371/journal.pone.0285570 (PMC10174535; doi:10.1371/journal.pone.0285570)
Supplement: S1 File — This provides further detail on the background of the measurement tools (S1 Table), cognitive tests (S2 Table), body composition (S3 Table), physical function tests (S4 Table) and caregiver questionnaire (S5 Table). Correlation coefficients between growth variable are also presented (S6 Table) and associations between the individual physical function tests (S7 Table) and between the individual cognitive function tests (S8 Table). Exploratory analysis between growth and physical function tests are shown (S9 Table), and also the exposures of schooling years and child socioemotional score on cognitive function tests (S10 Table). Exploratory associations between growth variables and cognitive function tests are presented (S11 Table). S1 Fig demonstrates the application of the SAHARAN Toolbox in photographs. S2 Fig portrays the associations explored between individual physical function tests and between individual cognitive function tests. S3 Fig shows a diagram exploring the associations of key growth variables on total physical function, and also HAZ and WAZ growth exposures on each individual physical function test. S4 Fig presents a diagram exploring the associations between exposures of years of schooling and child’s socioemotional support on each individual cognitive function test. S5 Fig demonstrates the associations between key growth variables as exposures on cognitive function tests as an outcome. (DOCX) [file pone.0285570.s001.docx]

**Supporting information**

Table of Contents

[Measures and constructs of interest 1](#_Toc117242600)

[Table S1: Tool selection 4](#_Toc117242601)

[Detailed methods 9](#_Toc117242602)

[**1.** **Cognitive function** 9](#_Toc117242603)

[2. Body composition and anthropometry 10](#_Toc117242604)

[3. Physical Function 11](#_Toc117242605)

[4. Caregiver questionnaire 12](#_Toc117242606)

[Supplementary tables 14](#_Toc117242607)

[Supplementary association tables 15](#_Toc117242608)

[Supplementary figures 18](#_Toc117242609)

[References 23](#_Toc117242610)

# **Measures and constructs of interest**

The SAHARAN toolbox combines measures of growth, body composition, cognitive and physical function to provide an overall assessment of child function. This new combined tool was designed to enable us to identify relationships between domains which are not usually measured simultaneously. Here we describe the different constructs of interest and the choice of measurement instruments used, based on the COSMIN study design checklist, and the methodology used for pre-testing and piloting, following principles previously described[1].

1. **School-aged Cognitive function**

Cognitive function is best assessed using multiple tools that measure distinct domains. Cognition is affected by multiple factors including undernutrition, nurturing, maternal and child interactions and stimulation[2]. Children in low-resource settings often have multiple exposures including socioeconomic adversity, undernutrition and lack of psychosocial support[3]. These may have differential impacts on cognitive domains, reinforcing the need to measure multiple domains, as described below.

- 1. **Child functional summary**

A measure of overall child function can determine difficulties in sight, hearing and mobility, as well as socioemotional areas including behaviour, learning and psychological functioning[4]. By focusing on what the child may have difficulty doing, this provides an overview that is complementary to specific areas of cognitive function.

- 1. **Cognitive processing**

Traditional intelligence testing methods relied on measuring *acquired knowledge*, such as intelligence quotient (IQ) testing. These tools were frequently developed in Western settings with high school enrolment rates: their use in low-resource settings is contentious, particularly in areas with variable schooling exposure[5]. Therefore, tools that measure *learning* *potential* are often preferred in low-resource environments[5]. These measure ‘cognitive processing’, which encapsulates the underlying processes and skills necessary to solve tasks, and are less sensitive to cultural and schooling bias[5, 6]. Hence a tool that measures cognitive processing across several domains of short and long-term learning memory, spatial reasoning and problem solving is preferred.

- 1. **Academic skills**

Complementary to cognitive processing, traditional measures of educational achievement are also important predictors for later academic and socioemotional function[7]:

1. Literacy: Early measures of language are indicative of future function including literacy, behaviour and socioemotional function[8]. Unless children learn to read at an early age, they cannot absorb more advanced skills and content that rely on reading, and typically fall behind in educational achievement[9]. Similarly, writing skills such as the child’s name[10] or dictation of words have also been demonstrated to be discriminatory for early-grade schooling exposure[11] and emergent literacy skills[10].
2. Mathematical skills: Mathematical literacy (including numeracy) is increasingly recognised as a core skill in everyday life[12]. Hence measuring early-age mathematical capabilities similarly predicts later engagement, learning and educational achievement[13].
   1. **Executive function**

Executive function includes universal skills such as inhibitory control, working memory, attention, and cognitive flexibility[14]. These skills enable children to plan, focus attention, set and achieve goals. They are highly related both to school readiness in young children[15] and later educational outcomes[16]. Executive function is also related both to socioemotional function[15] and academic skills[7, 15]. These goal-directed behaviours may be viewed as culturally universal skills[17] and therefore a tool that can specifically measure executive function may be included in future measurements.

- 1. **Behaviour and Socioemotional function**

Behavioural and emotional problems are common in children. Externalising behavioural problems such as attention deficit hyperactivity disorder may occur in up to 9% of preschool children in high-income settings[18] and this may be higher in some low-resource settings[19]. Child mental health (including behavioural measures) is increasingly recognised as an important measure affecting cognitive function[20]. Cognitive, academic, executive and socioemotional function are inter-related both in early childhood[21] and adolescence[22]. Therefore, this domain is also included and often measured through interview of caregivers or teachers[19, 23].

- 1. **Fine motor function**

Fine motor coordination is important for completion of many tasks, and has previously been identified to be reduced in stunted children, particularly those with worse academic function[24]. Reduced ability in fine motor function has also been associated with low birthweight and reduced socioemotional function[25], as well as reduced academic function[26]. This may be in part due to lags in development[27].

.

1. **School-aged physical function**

Systematic reviews have shown that child physical function and health are best assessed by combining separate measures of cardiovascular fitness, muscular strength and body composition[28, 29]. For example, cross-sectional data have shown that both cardiorespiratory fitness and muscular strength in children are associated with lower cardiovascular disease risk factors in later life (including lower blood pressure, blood lipids and adiposity)[28, 30]. There is also strong evidence that changes in muscular strength from childhood to adolescence are negatively associated with changes in overall adiposity[28]. Therefore, physical function measurements should include both cardiovascular fitness and muscular strength.

The physical function tests selected were similar to the ALPHA[29] and PREFIT[31] test batteries. The choice of tests was based on a systematic review of the literature,[32] combined with what had been previously piloted in LMIC (Kerac, personal communication). There is increasing evidence of physical activity being associated with improved cognitive ability, but this is mainly in higher income settings[33]. Physical fitness has also been described as a powerful marker of child health and future cardiovascular risk[30]. With the double burden of malnutrition, there is a rising trend of obesity and overweight children in sub- Saharan Africa, and increasing interest in methods to measure physical fitness[34].

1. **Growth**

There is emerging evidence that stunted growth in early life affects later height, body composition and physical parameters such as blood pressure[35, 36]. Body composition at school-age and adolescence (measured using body mass index, skinfold thickness and waist circumference) is associated with cardiovascular risk factors such as blood lipids and carotid artery narrowing[28]. There is also strong evidence indicating that a high BMI in childhood and adolescence increases the risk of death later in life[28]. A recent review highlighted that interventions that solely improve linear growth, are only weakly associated with cognitive function[37]. There is some intriguing emerging evidence that body composition at birth is associated with socioemotional function, with increasing lean mass improving socioemotional health and fat mass decreasing it[38]. This likely reflects maternal life history trade-offs among offspring[39]. Therefore growth measurements included both anthropometry and body composition.

## **Table S1: Tool selection**

| **Domain** | **Sub-domain** | **Tool** | **Measurement(s)** | **Validity** | **Reliability** | **Responsiveness** |
| --- | --- | --- | --- | --- | --- | --- |
| Cognitive Function | Cognitive Processing | Kaufmann Assessment Battery for Children (KABC-II) | KABC-II measures across four domains of sequential, planning, learning and simultaneous scales.  8 core subtests can be combined as the mental processing index (MPI) to provide a global measure | The KABC-II was originally developed and validated using a large sample in the USA[40]. It has since been widely used across Africa[41], demonstrating robust factor analysis in Uganda[42] and psychometric validity in rural South Africa[43]). The KABC-II group of cognitive tests show less bias to school exposure in low-income settings [43]. Minor adaptations to the KABC-II required for this rural Zimbabwe population have been described elsewhere{Piper, 2022 #1226}. | Reliability has been demonstrated in USA[40] and South Africa[44]. Recently, the QualiND model has demonstrated improved KABC-II monitoring and quality assurance using regular video review[45] across multiple countries and languages in Africa, including with a Shona translation in Zimbabwe[45] | Using KABC-II, a significant effect on cognition was detected with a nutrition intervention in South African children aged 6-11 years on two of the subtests[46] whilst in Ethiopia, 5 year olds with poorer growth also had worse KABC-II scores[47]. HIV positive children performed significantly worse than HIV-negative children in South Africa, Zimbabwe, Malawi and Uganda [48]. Similarly in separate studies in Burkina Faso, both stunted children [49] and those exposed to alcohol in pregnancy[49] performed significantly worse on KABC-II subtests. |
| Cognitive Function | Executive Function | Plus EF^[[1]](#footnote-2)^ | Inhibitory control: Hearts and Flowers (H&F)(note adapted to be stars and flowers for use in Africa)  Inhibit interference: MSIT  The flanker task requires (spatial) selective attention and executive control. | The *PLUS-EF* tablet-based executive function tool, is an open-source android-based cognitive assessment tool that has been validated for school-aged children[16]. It has been adapted for use in the *PLUS -EF* tablet tests in urban Kenya[50]. It measures executive function including cognitive flexibility and inhibition using different tasks, of which 3 were used: Multi-source interference test, stars and flowers and flanker test. These tasks have been adapted for use in the *PLUS -EF* tablet tests in Kenya after extensive piloting[50]. [Note this only got added later so data are not presented in this paper].  Similar tests have been widely used across childhood, although mainly in high-income settings[51]. The MSIT has been shown to reliably activate the cingulo-frontal-parietal (CFP) cognitive/attention network by functional MRI[52]. | Each individual test has shown reliability for an individual assessment situation, with quoted Cronbach alpha of MSIT 0.91 and H&F 0.81 [16].  A separate analysis of the Flanker task has shown good test-retest reliability and internal consistency, with Cronbach alpha >0.8[53]. | MSIT performance has been shown to improve with age and brain function mapped using functional MRI[54]. The MSIT has also previously demonstrated an effect of socioeconomic status, subjective social status and perceived stress on children’s executive function[55].  Hearts and Flowers has previously shown detrimental effects of moving home and socioeconomic status[56] as well as positive impacts of schooling exposure with time[57].  The Flanker test has previously showed improved selective attention with age in 4 to 6 year olds[58] and its inhibitory control is closely associated with school readiness[59]. |
| Cognitive Function | Fine motor | Finger tapping | Time to perform sequential finger tapping | *The Rapid Sequential Continuous Movements* was a sensitive measure of fine motor skills that was associated with stunting in children in Jamaica[24]. | During development, test-retest reliability was >0.78 and inter-observer agreements were above 0.96[60]. | An impact of stunting was shown, as well as strong associations with a schooling achievement test and intelligence quotient (IQ)[60]. |
| Cognitive Function | Academic function | School achievement test | Numeracy, literacy,  Writing ability | Numeracy was assessed using elements from the Early Grade Maths Assessment[9] and UNICEF Multi-Indicator Cluster Survey (MICS) Foundational Learning Module[61], which had been widely applied across Zimbabwe.  Literacy was assessed using reading elements from *The Early Grade Reading Assessment (EGRA),* which has been widely used across Africa to assess literacy[9].  Name writing ability has been shown to associated with emergent literacy skills[10]. | EGMA report reliability with Spearman’s rho of above 0.94 for number identification, discrimination and missing number subtests, with Cronbach alpha of 0.94, 0.82 and 0.58 respectively[13].  MICS used very similar questions with strong inter-rate reliability and agreement with EGRA and EGMA tests[62].  EGRA report reliability in Liberia of the 3 main elements used: letter identification, familiar word reading and non-word reading had Cronbach Alpha values of 0.78, 0.74 and 0.80 respectively[63]. | EGRA has been previously used to assess individual levels of literacy in Kenya[64]. EGRA has been used to monitor also early grade reading interventions[65].  The overall structure of the test, has been similarly piloted in Bangladesh and is being used to assess children followed up in the WASH Benefits trial [66] (Tofail, personal communication). |
|  | Socioemotional function | Strengths and Difficulties Questionnaire | Total difficulties score | *The Strengths and Difficulties Questionnaire (SDQ)* is a brief screening caregiver questionnaire for child mental health and behavioural problems from age 3-16 years. Both parent and teacher version have been shown to have construct and prediction validity in Holland[67]. It has been widely used in Africa[23] and found to be highly acceptable and applicable in sub-Saharan African settings [68]. | Parent-reported total difficulties scores gave a Cronbach alpha of >0.77 in a large sample in Holland, although sub-scores were lower (0.42 to 0.8) [67]. | This tool recently demonstrated the impact of LNS in similar aged children in Ghana[69]. |
| Cognitive Function | Sensory and overall function | WG UNICEF child functioning module (CFM) | Overall score | This tool was developed across multiple countries by UNICEF with extensive pretesting, cognitive interviewing and adaption[70, 71]. | The child functioning module was successfully performed in Mexico, Samoa and Serbia. It provided consistent prevalence rates similar to other tools using cut-offs describing ‘a lot of difficulty’ in functional domains or ‘daily’ levels of anxiety[72]. | The CFM has also been previously used in the SHINE cohort at age 2 years and demonstrated good agreement in comparison with functional screening using the Malawi Development Assessment Tool (MDAT) [73, 74]. |
| Growth | Body composition | Bioimpedance | Impedance index (relative lean mass)  Lean mass index,  Phase angle | *Bioimpedance (BIA)* measures tissue health and the proportion of lean mass using an imperceptible electrical signal between the hand and foot[75]. This has been widely used globally to assess malnutrition[76], and the technique has been validated and calibration equations derived using other body composition techniques such as deuterium dilution[77] in the Gambia. |  | Bioimpedance has been used to show accretion of lean mass in children recovering from severe acute malnutrition (SAM)[76]. It has also been previously used in the SHINE study and showed a reduction with stunting (unpublished data).  Lean mass correlates with organ size [78], improved neurodevelopment [79] and reduced metabolic risk[80]. An Ethiopian birth cohort study showed an association between lean mass at birth and socio-emotional function measured using the SDQ[38]. |
| Growth | Body composition | Skinfold thickness |  | *Skinfold thickness* measures the subcutaneous fat layer around the body and describes its distribution. Triceps and maximal calf skinfold thicknesses give a measure of peripheral fat, whilst subscapular skinfolds measure central fat. | Acceptable inter-observer agreement using technical error of measurement was <1mm, within the ChroSAM study[81]. | Fat mass provides short-term benefits for survival [82], but has longer-term metabolic health costs. Skinfold thickness and its distribution is a useful measure that reflects child malnutrition[76] and also as a marker for chronic disease risk[83]. They have also been previously used in the SHINE study and showed a reduction with stunting and HIV-exposure (unpublished data). |
| Growth | Anthropometry |  | Height,  Weight,  Head circumference,  Waist circumference,  Hip circumference,  Mid-upper arm circumference,  Calf circumference, | *2d) Anthropometry***:** Height and weight provide body mass index (BMI) which is an important marker of metabolic health[83], together with waist circumference[84]. Head circumference is a reliable measure of brain growth and previous nutritional deprivation, and is highly correlated with neurodevelopment[85]. Calf circumference and mid-upper arm circumference are complementary to skinfold thicknesses in providing insights into the quality of growth[86]. | Intra-observer technical error of measurement varied between 1 to 7 mm in the ChroSAM study[81]. |  |
| Growth | Knee-heel length |  | Knee-heel length | *Knee-heel (tibial) length* is a more sensitive measure of poor growth than leg length or stature and hence may be disproportionately reduced in stunting[87]. |  | There is emerging evidence that knee-heel length is a proxy for organ size, e.g. kidney in stunted children [88]. It has also been previously used in the SHINE study and showed a reduction with stunting and HIV-exposure (unpublished data). |
| Physical function | Strength | Handgrip strength | Average Handgrip strength | Handgrip strength is one of the core tests both within the ALPHA[29] and PREFIT[31] test batteries. This has been selected from a systematic review of the literature,[32] combined with what had been previously piloted in Malawi (Kerac, personal communication). | Previous studies had shown high reliability coefficients=0.97 and 0.98 for right and left hands, respectively, and no difference between test and retest[29, 89]. | *Handgrip strength* can be reduced in stunting[90] and in long-term assessments after acute malnutrition[86]. |
| Physical function | Strength | Broad jump | Distance jumped | Broad jump is one of the core tests both within the ALPHA[29] and PREFIT[31] test batteries. This was similarly recommended from systematic review of the literature,[32] as a validated test of core muscular fitness[91]. It had been previously piloted in Malawi (Kerac, personal communication). | The broad jump has demonstrated good criterion validity and reliability. It had the strongest association with a range of both lower body muscular strength tests (eg vertical jump, squat jump and countermovement jump) and upper body strength tests (throw basketball, push ups and isometric strength)[91]. | *The broad jump* is a measure of truncal tone and fitness[90] and has been shown to be reduced in stunted children in South Africa[92]. |
| Physical function | Cardiovascular fitness | Shuttle Run Test | Level reached | *The 20 meter shuttle run test (SRT)* is used to measure physical and aerobic capacity[93]. It has been shown to have good criterion related validity for cardiorespiratory fitness in both adults and children[94]. The criterion validity of the 20 m shuttle run test has been shown to be superior to similar measures of cardiovascular fitness such as the mile walk/run test[29, 95]. | Reliability has been stated to be acceptable with no systematic bias[29, 95]. | Stunting was a strong predictor of decreased fitness in the beep test when applied in Kenya[96] |
| Blood Pressure |  |  |  | BP can be increased in stunting, particularly in combination with overweight[97]. |  | BP in 8 year-olds in Nepal was independently negatively associated with leg and kidney length[78], and is a marker of homeostatic reserve and later cardio-metabolic risk[98]. |

**Supplementary Table S1**: **Individual tools used within the SAHARAN toolbox.** Measures, validity, reliability and responsiveness are based on previous literature. Validity is defined as how applicable the tool is in measuring a functional construct. This includes face validity (how it looks to the population), cross-cultural validity, content validity (how it measures), construct and structural validity (if measures are based on consistent hypotheses and reflect appropriate functional domains).

Reliability is defined as how accurate the tool is in detecting genuine differences between individuals. This includes reproducibility, inter-rater and intra-rater measurement error and internal consistency between similar tests. Responsiveness is defined as how the tool detects changes over time, or in response to potential interventions or adversities.

# **Detailed methods**

### **Cognitive function**

|  | **TEST** | **DOMAIN** | **Primary measure** | **Secondary measures** | **RATIONALE** |
| --- | --- | --- | --- | --- | --- |
| **Cognitive**  **Function**  **(120 mins)** | **KABC-II** | Memory, spatial abilities, reasoning | Mental Processing Index (MPI) | Subtest scores | Measures cognitive processing: less schooling dependent |
|  | **School Achievement Test** | Total score | *Subtest scores* (numeracy, reading, writing*)* | Academic | Literacy & numeracy |
|  | **Fine motor** | Sequential finger tapping speed | Shortest time to complete finger tapping, average between both hands | Finger tapping: dominant & non-dominant shortest time | Fine motor |
|  | **Child socio-emotional questionnaire** | Home support | Total score |  | Child’s own perspective on home environment |
|  | **Washington Group Child function module (asked in caregiver questionnaire)** | Disability screening, including vision and hearing | Overall score | Hearing, vision, mobility problem subscale | Child functional abilities |
|  | **SDQ (asked in caregiver questionnaire)** | Socioemotional function | SDQ total score | SDQ subtest scores | Behaviour |

**Supplementary Table S2: Cognitive measurement tools and measures used in the SAHARAN toolbox.**

1.1 The 8 core subtests within the *Kaufman Assessment Battery for Children 2^nd^ Edition (KABC-II)* were the primary measure for cognition: Their scaled sum provided the mental processing index (MPI). It is available from [www.pearson.com](http://www.pearson.com). The subtests selected were Atlantis, Story completion, Number recall, Atlantis delayed, Rover, Triangles, Word Order and pattern reasoning[43]. Online training was kindly provided by data collectors and trainers on zoom based in Uganda, Zimbabwe[48] and South Africa[43].

1.2 *The School Achievement Test* design was guided from piloting and similar tests currently being used in school-aged follow-up of the WASH Benefits trial[66] (Tofail F, private communication). During the test, the child started with numeracy, then moved to reading letters, then syllables and then words in the child’s preferred language (79/80 children chose Shona, 1 chose English).

1.3 *Fine motor function was* guided by sharing training videos during pre-testing (Dr Chang-Lopez, private communication). The shortest time to complete the task of sequential finger tapping six times was the primary measure. The data collector demonstrated first, then the child did several practice sessions before doing it 3 times in a row to ensure they could perform sequential finger tapping without stopping. Then each child was timed to see how fast they did sequential finger tapping 6 times in a row. They repeated this for a total of 3 times on each hand and the fastest time was used for analysis. The average between the 2 fastest times for each hand was also calculated.

1.4 *The Child Socioemotional Questionnaire* investigated the child’s viewpoint on their socioemotional support within the home, together with one final question on food security. Four of the questions were previously used during an evaluation of a teacher’s program in Zambia (MPES)[99]. In addition, 2 questions were previously used during a pilot study for UNICEF called Healthy Promoting Schools (Dr Lisa Langhaug, private communication). All questions were cognitively interviewed and pre-tested before being used in the study.

1.5 The Washington Group / UNICEF child functioning module (WG) is an international screening tool used to identify children with disabilities[72]. This has been previously used in Zimbabwe and correlated with the validated Malawi Development Assessment Test (MDAT) score[74] at 24 months. It was used to assess caregiver-reported difficulties in hearing, vision, learning, communication or behaviour. A laminated pictorial Likert scale of answers was also used to help visualise responses.

*1.6 The Strengths and Difficulties Questionnaire (SDQ)* is a brief screening caregiver questionnaire for child mental health and behavioural problems from age 3-16 years. The SDQ asks the caregiver to describe their child’s behaviour during the past 6 months, using 25 questions. Responses were scored on a Likert scale from 0-2 to give a “Total difficulties score” which was a primary measure. The tool in English is free to download online (<https://www.sdqinfo.com/>). The total difficulties score was a primary measure. A laminated pictorial Likert scale of answers was used to assist the caregiver with choosing a response.

## **Body composition and anthropometry**

|  | **TEST** | **DOMAIN** | **Primary measure** | **Secondary measures** | **RATIONALE** |
| --- | --- | --- | --- | --- | --- |
| **Body composition (20 mins)** | **BIA** | Impedance of tissues (lean mass) | Lean mass index,  Phase angle | Impedance index | Quality of growth / metabolic health |
|  | **Knee-heel length** | Tibial growth | Median Knee-heel |  | Prioritization of growth |
|  | **Triceps, scapular, calf skinfolds** | Subcutaneous fat | Sum of skinfolds | Individual skinfolds,  Peripheral: central | Fat: peripheral c.f. central / metabolic health |
| **Anthropometry (15 mins)** | **Height,**  **weight** | Growth | BMI | HAZ, WAZ, | Metabolic health |
|  | **Head circ** | Brain volume | Head circumference | - | Prioritization of growth |
|  | **Waist circ, Hip circumference** | Abdominal size | Waist circ | Hip circumference | Metabolic health |
|  | **Calf circ, MUAC** | Peripheral fat / muscle | Calf circ, MUAC | - | Quality of growth |

**Supplementary Table S3: Tests of body composition and anthropometry in the SAHARAN toolbox**

*2a) Bioimpedance (BIA)* gives an impedance reading (*Z*), and Height^2^/Z gives the ‘impedance index’, which is a relative measure of lean mass within the sample. However, this requires a population-specific equation to provide the actual lean mass using another body composition technique[77]. The Lean mass index (1/Z) avoids the need for any equation and can be visualised as expressing variability in the lean mass component of body mass index, ie lean mass index expressed in the same kg/m^2^ units. Note that 1/Z is expressed in abstract units (1/Ohms), but the variability in 1/Z and the variability in lean mass index differ only in terms of the constants used to convert abstract to physical values[100]. This makes 1/Z highly effective at ranking variability in lean mass index. BIA was measured in recumbent children using the Bodystat 1500 MDD instrument (BodyStat, Isle of Man, UK). Electrodes were attached to the right hand and foot and BIA measurements performed with standard criteria to ensure repeatability. The mean of the two readings was used for each BIA measurement. Data were excluded if the phase angle was more than 8 degrees[101], or Z had poor repeatability (> 6 Ohms difference). BIA assessments were tolerated extremely well by children in the study.

*2b) Skinfold thickness* was measured to the nearest 0.2mm using a skinfold caliper (Holtain, Crosswell, Wales) and the median of 3 readings used. These were similarly tolerated extremely well by children in the study. All measurements were performed on the right side.

*2c) Knee-heel (tibial) length*: The right-sided median tibial length was assessed using a commercial knemometer (weighandmeasures.com, Olney, USA).

*2d) Anthropometry***:** Height was measured using a Shorrboard (Weighandmeasure, USA), weight using portable scales (Seca, Germany) and circumferences using anthropometry tape (Weighandmeasure, USA).

## **Physical Function**

|  | **TEST** | **DOMAIN** | **Primary measure** | **Secondary measures** | **RATIONALE** |
| --- | --- | --- | --- | --- | --- |
| **Physical**  **Function (30 mins)** | **Grip strength** | Lean muscle both hand | Highest grip strength, average in both hands: (**a)** | Dominant and non-dominant hand strength | Lean muscle: hand |
|  | **Broad jump** | Truncal muscles | Full distance: (**b)** |  | Lean muscle: trunk |
|  | **20m Shuttle run test (SRT)** | Physical Fitness, | Level achieved in shuttle run: (**c)** |  | Stamina, |
|  | **Total physical score (from grip strength, broad jump and SRT)** | ***Strength & fitness*** | Sum of 3 standardised scores above:  **(TPS) = a+b+c** |  | Composite physical function score |
|  | **Hemoglobin** | Anaemia | Hb |  | Physical fitness |
|  | **BP** | Fitness | Resting Systolic, diastolic bp | Pulse pressure, BP 1 minute after exercise | Cardiovascular fitness |
|  |  |  |  |  |  |

**Supplementary Table S4: Physical function measurements used in the SAHARAN toolbox**

*3a) Handgrip strength:* The Takei dynamometer was selected, as it was shown to have the highest criterion-related validity and reliability[102]. The dynamometer’s handgrip size was adjusted appropriately for the handspan[89]. It has also been reported that use with the elbow extended provides the most appropriate positioning[102]. Therefore, the child stood and held the Takei dynamometer vertically down and squeezed as hard as they could for up to 5 seconds. After a suitable break, they repeated this three times for each hand and the maximum value used for analysis.

*3b) The broad jump:* The child stands behind a line marked on the ground with feet slightly apart. A two-foot take-off and landing is used where the child swings the arms and bends the knees to provide forward drive, with the research nurse demonstrating first. The distance jumped is defined from the take-off line to the nearest point of contact on the landing (back of the heels), with the longest jump of 3 attempts used in analysis.

*3c) Shuttle run test (SRT):* A 20 meter tape measure is placed and the child runs repeatedly between each end, arriving before the beep, with increasingly shortened time gaps between beeps. A Bluetooth speaker is connected to the ODK tablet using the “Beep test” free android app (Beeptest, Ruval Enterprises, Canada). Once the child misses the beep three times in a row or stops running, the child then withdraws. The test provides a valid and reliable prediction of the VO_2max_[103], the maximum rate at which the body uses oxygen during exercise. The feasibility of heart rate monitoring using wearable wrist (Fitbit Charge, UK) and chest-based (Polar, UK) heart rate monitors during exercise was also explored[104]. *A composite total physical score (TPS)* was calculated based on the standardised results of highest handgrip score, furthest broad jump and highest level on 20m beep test to give an overall measure of physical function.

*3d) Blood pressure* (BP) was initially measured using an automated sphygmomanometer (Omron, Milton Keynes, UK) and then manual blood sphygmomanometer (Medisave, UK).

*3e) Haemoglobin* was measured (Hemocue) by a finger prick test as a potentially important contributor to physical fitness[86] and cognitive outcomes.

## **Caregiver questionnaire**

In parallel with the child measurements, a detailed caregiver questionnaire was also administered to measure household demographics, previous adversities and contemporary factors associated with child growth and function.

|  | **QUESTIONNAIRE** | **DOMAIN** | **Primary measure** | **Secondary measures** | **RATIONALE** |
| --- | --- | --- | --- | --- | --- |
| **Caregiver questionnaire (90 mins)** | **Demographics** | Household composition | Main caregiver | Years of schooling |  |
|  | **Socioeconomic status** | SES score | Overall score | - | Socio-economic status |
|  | **SDQ** | Socioemotional function | SDQ total score | SDQ subtest score | Behaviour |
|  | **Schooling & COVID impact** | School engagement & attendance | Years of schooling | Attendance  Alternative learning | Education |
|  | **Washington Group Child function module** | Disability screening, including vision and hearing | Overall score | Hearing, vision, mobility problem subscale | Child functional abilities |
|  | **Child adversity scale** | Adversities | Overall score |  | Measure of accumulated adversities |
|  | **Child parent relationship scale** | Caregiver’s relationship with child | Overall score |  | Nurturing |
|  | **MICS Child discipline score** | Caregiver’s relationship with child | Overall score |  | Nurturing |
|  | **EPDS** | Maternal depression | Overall score | - | Depression |
|  | **HFIAS, HDDS** | Food insecurity | HDDS score  HFIAS score |  | Food insecurity |
|  | **HWISE, Water access** | Water insecurity & access | HWISE score, | water volume | Water insecurity |

**Supplementary Table S5: Caregiver questionnaire sections.** MICS: Multi-indicator cluster survey (UNICEF), EPDS: Edinburgh postnatal depression score, HFIAS: Household Food Insecurity Access Scale, HDDS: Household Dietary Diversity scale, HWISE: Household Water Insecurity Experiences Scale.

*Demographics* related to household composition and the primary caregiver. *Socioeconomic status* was measured using a wealth index previously developed for the region of the study[105]. *Schooling exposure and COVID impact* were also asked. *The Washington Group / UNICEF Child Functioning module*[4, 106] provides a screening tool for functional difficulties in hearing, vision, communication, comprehension, learning, mobility and emotions using a rating scale. *The child adversities index* screened for major life adversities associated with reduced child development since birth[107, 108]. These questions were carefully piloted and selected for a region with minimal social services support. Key elements of nurturing were measured using the Child-Parent Relationship scale[109] and MICS Child Discipline questionnaire[61, 110]. Caregiver depression was measured using local translations of The Edinburgh Postnatal Depression score (EPDS), which has been validated and extensively used in this region[111]. Food insecurity was measured using the Household Food Insecurity Access Scale (HFIAS)[112] and Household Dietary Diversity Scale (HDDS)[113]. The Household Water Insecurity Experiences Scale (HWISE)[114] measured water insecurity.

# **Supplementary tables**

**S6 Table: Pearson correlation coefficients between growth variables**. Those with correlations >0.79 are highlighted in grey[115] and selected variables are in bold. bmiz: BMI z-score, head circ: head circumference, muac: mid-upper arm circumference, waist circ: waist circumference, hip circ: hip circumference, calf circ: calf circumference, LMI: lean mass index, Imp Index: impedance index, total SFT: total skinfold thickness, Triceps SFT: triceps skinfold thickness, subscapular SFT: subscapular skinfold thickness, calf SFT: calf skinfold thickness, Hb: haemoglobin.

## Supplementary association tables

Additional associations within the SAHARAN toolbox were measured by least squares linear regression analysis as described below:

| Dependent variable | Independent variable | Regression Coefficient | 95% CI | P value |
| --- | --- | --- | --- | --- |
| Grip strength / N | **Max broad jump / cm** | **0.06** | **{0.03, 0.09}** | **<0.001** |
| Shuttle run test level | **Grip strength / N** | **0.19** | **{0.07, 0.3}** | **<0.001** |
| Shuttle run test level | Broad jump max / cm | 0.01 | {-0.01, 0.03} | 0.22 |

**S7 Table: Exploring the associations between physical function tests.**

| Dependent variable | Independent variable | Regression Coefficient | 95% CI | P value |
| --- | --- | --- | --- | --- |
| MPI | **SAT** | **0.19** | **{0.09, 0.29}** | **<0.001** |
| MPI | SDQ total | -0.19 | {-0.59, 0.28} | 0.47 |
| MPI | Fine motor | -0.4 | {-0.84, 0.03} | 0.07 |
| SAT | Fine motor | -0.57 | {-1.58, 0.45} | 0.27 |
| SAT | SDQ total | -0.81 | {-1.81, 0.18} | 0.11 |
| Fine motor | SDQ total | 0.07 | {-0.25, 0.39} | 0.47 |

**S8 Table: Exploring the associations between cognitive function tests.** MPI: Mental processing index (KABC-II total scaled for relative age). SAT: School Achievement Test, SDQ: Strengths and difficulties questionnaire, Fine motor: Fine motor test score.

| Dependent variable | Independent variable | Regression Coefficient | 95% CI | P value |
| --- | --- | --- | --- | --- |
| Total physical score | **WAZ** | **1.08** | **{0.55, 1.61}** | **<0.001** |
| Total physical score | **HAZ** | **1.29** | **{0.75, 1.82}** | **<0.001** |
| Total physical score | **Phase angle** | **1.14** | **{0.33, 1.95}** | **0.01** |
| Total physical score | **LMI** | **0.5** | **{0.16, 0.83}** | **<0.001** |
| Total physical score | **Head circ** | **0.4** | **{0.13, 0.66}** | **<0.001** |
| Total physical score | **MUAC** | **0.44** | **{0.07, 0.82}** | **0.02** |
| Total physical score | **Calf circ** | **0.34** | **{0.08, 0.6}** | **0.01** |
| Total physical score | Waist circ | 0.13 | {-0.03, 0.29} | 0.12 |
| Total physical score | Total SFT | -0.07 | {-0.19, 0.04} | 0.2 |
| Total physical score | Hb | 0.03 | {-0.42, 0.49} | 0.88 |
| Shuttle run test level | WAZ | 0.3 | {-0.01, 0.61} | 0.05 |
| Max broad jump | **WAZ** | **5.22** | **{1.17, 9.26}** | **0.01** |
| Grip strength | **WAZ** | **1.17** | **{0.65, 1.69}** | **<0.001** |
| Shuttle run test level | **HAZ** | **0.39** | **{0.07, 0.71}** | **0.02** |
| Max broad jump | **HAZ** | **6.72** | **{2.57, 10.87}** | **<0.001** |
| Grip strength | **HAZ** | **1.24** | **{0.7, 1.78}** | **<0.001** |

**S9 Table: Exploring the associations between growth variables and physical function** head circ: head circumference, muac: mid-upper arm circumference, waist circ: waist circumference, calf circ: calf circumference, LMI: lean mass index, total SFT: total skinfold thickness, Hb: haemoglobin.

| Dependent variable | Independent variable | Regression Coefficient | 95% CI | P value |
| --- | --- | --- | --- | --- |
| MPI | Years of Schooling | 2.65 | {-0.11, 5.41} | 0.06 |
| SAT | **Years of Schooling** | **18.71** | **{13.99, 23.44}** | **<0.001** |
| SDQ total | Years of Schooling | -0.70 | {-2.15, 0.75} | 0.34 |
| Fine motor | Years of Schooling | -0.9 | {-3.21, 1.42} | 0.44 |
| MPI | Child Socioemotional | 2.42 | {-0.09 4.92} | 0.06 |
| SAT | **Child Socioemotional** | **8.2** | **{2.87, 13.53}** | **<0.001** |
| SDQ total | Child Socioemotional | 0.07 | {-1.31, 1.45} | 0.92 |
| Fine motor | Child Socioemotional | -0.41 | {-2.79, 1.98} | 0.73 |

**S10 Table**: **Exploring the associations between exposures of a) years of schooling and b) child socioemotional score on cognitive function tests.** Child Socioemotional: Child’s perceived socioemotional support score, MPI: Mental processing index (KABC-II total scaled for relative age), SAT: School Achievement Test, SDQ: Strengths and difficulties questionnaire where negative scores represent improved function, Fine motor: Fine motor test score (time taken to complete finger tapping test in seconds, so negative scores represent improved function).

| Dependent variable | Independent variable | Regression Coefficient | 95% CI | P value |
| --- | --- | --- | --- | --- |
| MPI | WAZ | -0.43 | {-2.9, 2.05} | 0.73 |
| SAT | WAZ | 3.45 | {-2.16, 9.07} | 0.22 |
| SDQ total | WAZ | -0.31 | {-1.59, 0.97} | 0.63 |
| Fine motor | WAZ | -0.24 | {-2.24, 1.77} | 0.81 |
| MPI | HAZ | 0.65 | {-1.95, 3.24} | 0.62 |
| SAT | HAZ | 1.28 | {-4.73, 7.29} | 0.67 |
| SDQ total | HAZ | -0.86 | {-2.20, 0.46} | 0.20 |
| Fine motor | HAZ | -0.24 | {-2.24, 1.77} | 0.81 |
| MPI | Phase angle | 1.76 | {-1.84, 5.36} | 0.33 |
| SAT | Phase angle | 5.3 | {-2.83 13.43} | 0.2 |
| SDQ total | Phase angle | -0.74 | {-2.61, 1.12} | 0.43 |
| Fine motor | **Phase angle** | **-3.46** | **{-6.37, -0.55}** | **0.02** |
| MPI | LMI | 0 | {-1.52, 1.52} | 1 |
| SAT | LMI | -0.49 | {-3.95, 2.98} | 0.78 |
| SDQ total | LMI | 0.31 | {-0.47, 1.10} | 0.43 |
| Fine motor | LMI | 0.17 | {-1, 1.35} | 0.77 |
| MPI | Hb | -1.08 | {-3.02, 0.86} | 0.27 |
| SAT | Hb | 0.22 | {-4.17, 4.61} | 0.92 |
| SDQ total | Hb | -0.42 | {-1.43, 0.59} | 0.40 |
| Fine motor | Hb | -0.67 | {-1.98, 0.63} | 0.30 |
| MPI | Head circ | 0.47 | {-0.71, 1.66} | 0.43 |
| SAT | Head circ | -0.42 | {-3.11, 2.27} | 0.76 |
| SDQ total | Head circ | 0.51 | {-0.09, 1.11} | 0.10 |
| Fine motor | Head circ | 0.13 | {-0.76, 1.02} | 0.76 |
| MPI | Total SFT | 0.03 | {-0.46, 0.52} | 0.9 |
| SAT | Total SFT | 1.44 | {0.38, 2.49} | 0.01 |
| SDQ total | Total SFT | -0.07 | {-0.32, 0.19} | 0.60 |
| Fine motor | Total SFT | -0.02 | {-0.53, 0.5} | 0.95 |
| MPI | Waist circ | -0.5 | {-1.19, 0.19} | 0.16 |
| SAT | Waist circ | -0.23 | {-1.88, 1.42} | 0.78 |
| SDQ total | Waist circ | -0.03 | {-0.39, 0.33} | 0.88 |
| Fine motor | Waist circ | 0.11 | {-0.45, 0.68} | 0.69 |
| MPI | MUAC | -0.71 | {-2.36, 0.95} | 0.4 |
| SAT | **MUAC** | **4.67** | **{1.04, 8.29}** | **0.01** |
| SDQ total | MUAC | -0.22 | {-1.08, 0.64} | 0.61 |
| Fine motor | MUAC | -0.53 | {-2.01, 0.94} | 0.47 |
| MPI | Calf circ | -0.16 | {-1.3, 0.99} | 0.79 |
| SAT | Calf circ | 2.43 | {-0.09, 4.96} | 0.06 |
| SDQ total | Calf circ | -0.27 | {-0.86, 0.31} | 0.36 |
| Fine motor | Calf circ | -0.05 | {-0.98, 0.88} | 0.91 |

**S11 Table: Exploring the associations between growth variables and cognitive function** head circ: head circumference, muac: mid-upper arm circumference, waist circ: waist circumference, calf circ: calf circumference, LMI: lean mass index, total SFT: total skinfold thickness, Hb: haemoglobin, MPI: Mental processing index scaled score, SAT: School achievement test total score, SDQ: Strengths and difficulties questionnaire, Fine motor: Time taken to complete sequential finger tapping.

# **Supplementary figures**

**S1 Fig. Application of the SAHARAN toolbox.** A: Portable handwashing station and role play. B: tent arrangement for caregiver and child (here using a tree for shade). C: Child cognitive measurement using the School Achievement Test (SAT). D: Child body composition measurement using Bioimpedance Impedance Analysis (BIA).

**S2 Fig. Exploring the associations within individual physical function tests and cognitive function tests.**

**S3 Fig. Exploring the associations between a) key growth variables as exposures on total physical function as an outcome; b) haz and waz as exposures on each individual physical function test as an outcome.**

**S4 Fig. Exploring the associations between a) exposures of years of schooling and b) child’s perceived socioemotional support score on the outcome of cognitive function tests.**

**S5 Fig. Exploring the associations between key growth variables as exposures on cognitive function tests as an outcome (red arrows).**

# **References**

1. Boateng GO, Neilands TB, Frongillo EA, Melgar-Quiñonez HR, Young SL. Best Practices for Developing and Validating Scales for Health, Social, and Behavioral Research: A Primer. Frontiers in public health. 2018;6:149-. doi: 10.3389/fpubh.2018.00149. PubMed PMID: 29942800.

2. Worku BN, Abessa TG, Wondafrash M, Vanvuchelen M, Bruckers L, Kolsteren P, et al. The relationship of undernutrition/psychosocial factors and developmental outcomes of children in extreme poverty in Ethiopia. BMC Pediatrics. 2018;18(1):45. doi: 10.1186/s12887-018-1009-y.

3. Walker SP, Wachs TD, Gardner JM, Lozoff B, Wasserman GA, Pollitt E, et al. Child development: risk factors for adverse outcomes in developing countries. Lancet (London, England). 2007;369(9556):145-57. Epub 2007/01/16. doi: 10.1016/s0140-6736(07)60076-2. PubMed PMID: 17223478.

4. Loeb M, Mont D, Cappa C, De Palma E, Madans J, Crialesi R. The development and testing of a module on child functioning for identifying children with disabilities on surveys. I: Background. Disabil Health J. 2018;11(4):495-501. Epub 07/18. doi: 10.1016/j.dhjo.2018.06.005. PubMed PMID: 30054226.

5. Alcock KJ, Holding PA, Mung'ala-Odera V, Newton CRJC. Constructing Tests of Cognitive Abilities for Schooled and Unschooled Children. Journal of Cross-Cultural Psychology. 2008;39(5):529-51. doi: 10.1177/0022022108321176.

6. Sternberg RJ, Grigorenko EL. Intelligence and culture: how culture shapes what intelligence means, and the implications for a science of well-being. Philos Trans R Soc Lond B Biol Sci. 2004;359(1449):1427-34. doi: 10.1098/rstb.2004.1514. PubMed PMID: 15347533.

7. Wolf S, McCoy DC. The role of executive function and social-emotional skills in the development of literacy and numeracy during preschool: a cross-lagged longitudinal study. Dev Sci. 2019;22(4):e12800. Epub 2019/01/23. doi: 10.1111/desc.12800. PubMed PMID: 30666761.

8. McKean C, Reilly S, Bavin EL, Bretherton L, Cini E, Conway L, et al. Language Outcomes at 7 Years: Early Predictors and Co-Occurring Difficulties. Pediatrics. 2017;139(3):e20161684. doi: 10.1542/peds.2016-1684.

9. Gove A, Brunette T, Bulat J, Carrol B, Henny C, Macon W, et al. Assessing the Impact of Early Learning Programs in Africa. New Dir Child Adolesc Dev. 2017;2017(158):25-41. doi: 10.1002/cad.20224. PubMed PMID: 29243385.

10. Puranik CS, Lonigan CJ. Name-writing proficiency, not length of name, is associated with preschool children's emergent literacy skills. Early Child Res Q. 2012;27(2):284-94. Epub 2011/09/21. doi: 10.1016/j.ecresq.2011.09.003. PubMed PMID: 22523450.

11. Nicolau CC, Navas ALGP. Avaliação das habilidades preditoras do sucesso de leitura em crianças de 1º e 2º anos do ensino fundamental. Revista CEFAC. 2015;17:917-26.

12. Steen L. Mathematics and democracy: The case for quantitative literacy. 2001.

13. Platas LM, Ketterlin-Gellar L, Brombacher A, Sitabkhan Y. Early Grade Maths Assessment Toolkit (EGMA). RTI. <https://shared.rti.org/content/early-grade-mathematics-assessment-egma-toolkit2014>.

14. Harvard University. Executive Function and Self Regulation 2020 [cited 2020 21 December]. Available from: <https://developingchild.harvard.edu/science/key-concepts/executive-function/>.

15. Willoughby MT, Piper B, Oyanga A, Merseth King K. Measuring executive function skills in young children in Kenya: Associations with school readiness. Dev Sci. 2019;22(5):e12818. Epub 2019/02/20. doi: 10.1111/desc.12818. PubMed PMID: 30779264.

16. Obradović J, Sulik MJ, Finch JE, Tirado-Strayer N. Assessing students' executive functions in the classroom: Validating a scalable group-based procedure. Journal of Applied Developmental Psychology. 2018;55:4-13. doi: 10.1016/j.appdev.2017.03.003.

17. Obradović J, Willoughby MT. Studying Executive Function Skills in Young Children in Low- and Middle-Income Countries: Progress and Directions. Child Development Perspectives. 2019;13(4):227-34. doi: <https://doi.org/10.1111/cdep.12349>.

18. Froehlich TE, Lanphear BP, Epstein JN, Barbaresi WJ, Katusic SK, Kahn RS. Prevalence, recognition, and treatment of attention-deficit/hyperactivity disorder in a national sample of US children. Arch Pediatr Adolesc Med. 2007;161(9):857-64. Epub 2007/09/05. doi: 10.1001/archpedi.161.9.857. PubMed PMID: 17768285.

19. Kariuki SM, Abubakar A, Murray E, Stein A, Newton CRJC. Evaluation of psychometric properties and factorial structure of the pre-school child behaviour checklist at the Kenyan Coast. Child and adolescent psychiatry and mental health. 2016;10(1):1. doi: 10.1186/s13034-015-0089-9.

20. Dias NM, Seabra AG. Mental Health, Cognition and Academic Performance in the 1st Year of Elementary Education. Psico-USF. 2020;25:467-79.

21. Ferrier DE, Bassett HH, Denham SA. Relations between executive function and emotionality in preschoolers: Exploring a transitive cognition-emotion linkage. Frontiers in psychology. 2014;5:487-. doi: 10.3389/fpsyg.2014.00487. PubMed PMID: 24904500.

22. Vandenbroucke L, Weeda W, Lee N, Baeyens D, Westfall J, Figner B, et al. Heterogeneity in Cognitive and Socio-Emotional Functioning in Adolescents With On-Track and Delayed School Progression. Frontiers in psychology. 2018;9(1572). doi: 10.3389/fpsyg.2018.01572.

23. Hoosen N, Davids EL, de Vries PJ, Shung-King M. The Strengths and Difficulties Questionnaire (SDQ) in Africa: a scoping review of its application and validation. Child and adolescent psychiatry and mental health. 2018;12. doi: 10.1186/s13034-017-0212-1.

24. Chang SM, Walker SP, Grantham‐Mcgregor S, Powell CA. Early childhood stunting and later fine motor abilities. Developmental Medicine & Child Neurology. 2010;52(9):831-6. doi: 10.1111/j.1469-8749.2010.03640.x.

25. Breslau N, Chilcoat HD, Johnson EO, Andreski P, Lucia VC. Neurologic soft signs and low birthweight: their association and neuropsychiatric implications. Biol Psychiatry. 2000;47(1):71-9. Epub 2000/01/29. doi: 10.1016/s0006-3223(99)00131-6. PubMed PMID: 10650451.

26. Batstra L, Neeleman J, Hadders-Algra M. The neurology of learning and behavioural problems in pre-adolescent children. Acta psychiatrica Scandinavica. 2003;108(2):92-100. Epub 2003/06/26. doi: 10.1034/j.1600-0447.2003.00127.x. PubMed PMID: 12823165.

27. Blondis TA, Snow JH, Accardo PJ. Integration of soft signs in academically normal and academically at-risk children. Pediatrics. 1990;85(3 Pt 2):421-5. Epub 1990/03/01. PubMed PMID: 2304803.

28. Ruiz JR, Castro-Piñero J, Artero EG, Ortega FB, Sjöström M, Suni J, et al. Predictive validity of health-related fitness in youth: a systematic review. British journal of sports medicine. 2009;43(12):909-23. Epub 2009/01/23. doi: 10.1136/bjsm.2008.056499. PubMed PMID: 19158130.

29. Ruiz JR, Castro-Piñero J, España-Romero V, Artero EG, Ortega FB, Cuenca MM, et al. Field-based fitness assessment in young people: the ALPHA health-related fitness test battery for children and adolescents. British journal of sports medicine. 2011;45(6):518-24. Epub 2010/10/22. doi: 10.1136/bjsm.2010.075341. PubMed PMID: 20961915.

30. Ortega FB, Ruiz JR, Castillo MJ, Sjöström M. Physical fitness in childhood and adolescence: a powerful marker of health. International journal of obesity (2005). 2008;32(1):1-11. Epub 2007/11/29. doi: 10.1038/sj.ijo.0803774. PubMed PMID: 18043605.

31. Cadenas-Sanchez C, Martinez-Tellez B, Sanchez-Delgado G, Mora-Gonzalez J, Castro-Piñero J, Löf M, et al. Assessing physical fitness in preschool children: Feasibility, reliability and practical recommendations for the PREFIT battery. Journal of science and medicine in sport. 2016;19(11):910-5. Epub 2016/03/08. doi: 10.1016/j.jsams.2016.02.003. PubMed PMID: 26947061.

32. Ortega FB, Cadenas-Sánchez C, Sánchez-Delgado G, Mora-González J, Martínez-Téllez B, Artero EG, et al. Systematic review and proposal of a field-based physical fitness-test battery in preschool children: the PREFIT battery. Sports Med. 2015;45(4):533-55. Epub 2014/11/06. doi: 10.1007/s40279-014-0281-8. PubMed PMID: 25370201.

33. Donnelly JE, Hillman CH, Castelli D, Etnier JL, Lee S, Tomporowski P, et al. Physical Activity, Fitness, Cognitive Function, and Academic Achievement in Children: A Systematic Review. Medicine and science in sports and exercise. 2016;48(6):1197-222. doi: 10.1249/MSS.0000000000000901. PubMed PMID: 27182986.

34. Klingberg S, Draper CE, Micklesfield LK, Benjamin-Neelon SE, van Sluijs EMF. Childhood Obesity Prevention in Africa: A Systematic Review of Intervention Effectiveness and Implementation. International journal of environmental research and public health. 2019;16(7):1212. doi: 10.3390/ijerph16071212. PubMed PMID: 30987335.

35. Rolfe EDL, França GVAd, Vianna CA, Gigante DP, Miranda JJ, Yudkin JS, et al. Associations of stunting in early childhood with cardiometabolic risk factors in adulthood. PLOS ONE. 2018;13(4):e0192196. doi: 10.1371/journal.pone.0192196.

36. Wells JCK, Devakumar D, Grijalva-Eternod CS, Manandhar DS, Costello A, Osrin D. Blood pressure and the capacity-load model in 8-year-old children from Nepal: Testing the contributions of kidney size and intergenerational effects. Am J Hum Biol. 2016;28(4):555-65. doi: 10.1002/ajhb.22829.

37. Prado EL, Larson LM, Cox K, Bettencourt K, Kubes JN, Shankar AH. Do effects of early life interventions on linear growth correspond to effects on neurobehavioural development? A systematic review and meta-analysis. The Lancet Global Health. 2019;7(10):e1398-e413. doi: 10.1016/S2214-109X(19)30361-4.

38. Abera M, Tesfaye M, Hanlon C, Admassu B, Girma T, Wells JC, et al. Body Composition during Early Infancy and Mental Health Outcomes at 5 Years of Age: A Prospective Cohort Study of Ethiopian Children. The Journal of pediatrics. 2018;200:225-31. doi: <https://doi.org/10.1016/j.jpeds.2018.04.055>.

39. Wells JCK. Life history trade-offs and the partitioning of maternal investment. Evolution, Medicine, and Public Health. 2018;2018(1):153-66. doi: 10.1093/emph/eoy014.

40. Kaufmann A, Kaufmann N. Kaufman Assessment Battery for Children 2nd edition. In: PsychCorp, editor. 2nd Edition: Pearson; 2004.

41. Semrud-Clikeman M, Romero RAA, Prado EL, Shapiro EG, Bangirana P, John CC. Selecting measures for the neurodevelopmental assessment of children in low- and middle-income countries. Child neuropsychology : a journal on normal and abnormal development in childhood and adolescence. 2017;23(7):761-802. Epub 09/09. doi: 10.1080/09297049.2016.1216536. PubMed PMID: 27609060.

42. Bangirana P, Seggane M, Allebeck P, Giordani B, John CC, Opoka OR, et al. A preliminary examination of the construct validity of the KABC-II in Ugandan children with a history of cerebral malaria. African health sciences. 2009;9(3):186-92. Epub 2010/07/01. PubMed PMID: 20589149; PubMed Central PMCID: PMCPMC2887024.

43. Mitchell JM, Tomlinson M, Bland RM, Houle B, Stein A, Rochat TJ. Confirmatory factor analysis of the Kaufman assessment battery in a sample of primary school-aged children in rural South Africa. South African Journal of Psychology. 2018;48(4):434-52. doi: 10.1177/0081246317741822.

44. Mitchell JM, Tomlinson M, Rochat DT. Psychometric evaluation of the Kaufman Assessment Battery for Children, Second Edition (KABC-II) in rural South Africa: Stellenbosch; 2015.

45. Ruisenor-Escudero H, Familiar I, Nyakato M, Kutessa A, Namukooli J, Ssesanga T, et al. Building capacity in neurodevelopment assessment of children in sub-Saharan Africa: A quality assurance model to implement standardized neurodevelopment testing. Child neuropsychology : a journal on normal and abnormal development in childhood and adolescence. 2019;25(4):466-81. doi: 10.1080/09297049.2018.1497588.

46. Taljaard C, Covic NM, van Graan AE, Kruger HS, Smuts CM, Baumgartner J, et al. Effects of a multi-micronutrient-fortified beverage, with and without sugar, on growth and cognition in South African schoolchildren: a randomised, double-blind, controlled intervention. The British journal of nutrition. 2013;110(12):2271-84. Epub 2013/07/05. doi: 10.1017/s000711451300189x. PubMed PMID: 23823584.

47. Bogale A, Stoecker BJ, Kennedy T, Hubbs-Tait L, Thomas D, Abebe Y, et al. Nutritional status and cognitive performance of mother-child pairs in Sidama, Southern Ethiopia. Maternal & child nutrition. 2013;9(2):274-84. Epub 2011/08/03. doi: 10.1111/j.1740-8709.2011.00345.x. PubMed PMID: 21806779.

48. Boivin MJ, Barlow-Mosha L, Chernoff MC, Laughton B, Zimmer B, Joyce C, et al. Neuropsychological performance in African children with HIV enrolled in a multisite antiretroviral clinical trial. AIDS (London, England). 2018;32(2):189-204. doi: 10.1097/QAD.0000000000001683.

49. Sanou AS, Diallo AH, Holding P, Nankabirwa V, Engebretsen IMS, Ndeezi G, et al. Association between stunting and neuro-psychological outcomes among children in Burkina Faso, West Africa. Child and adolescent psychiatry and mental health. 2018;12(1):30. doi: 10.1186/s13034-018-0236-1.

50. Kariger P, Sulik MJ, Obradović J. Piloting of Plus EF in Kenya. 2019.

51. Davidson MC, Amso D, Anderson LC, Diamond A. Development of cognitive control and executive functions from 4 to 13 years: evidence from manipulations of memory, inhibition, and task switching. Neuropsychologia. 2006;44(11):2037-78. Epub 2006/03/31. doi: 10.1016/j.neuropsychologia.2006.02.006. PubMed PMID: 16580701.

52. Bush G, Shin LM. The Multi-Source Interference Task: an fMRI task that reliably activates the cingulo-frontal-parietal cognitive/attention network. Nat Protoc. 2006;1(1):308-13. Epub 2007/04/05. doi: 10.1038/nprot.2006.48. PubMed PMID: 17406250.

53. Wöstmann NM, Aichert DS, Costa A, Rubia K, Möller H-J, Ettinger U. Reliability and plasticity of response inhibition and interference control. Brain and Cognition. 2013;81(1):82-94. doi: <https://doi.org/10.1016/j.bandc.2012.09.010>.

54. Liu Y, Angstadt M, Taylor SF, Fitzgerald KD. The typical development of posterior medial frontal cortex function and connectivity during task control demands in youth 8–19years old. NeuroImage. 2016;137:97-106. doi: <https://doi.org/10.1016/j.neuroimage.2016.05.019>.

55. Ursache A, Noble KG, Blair C. Socioeconomic Status, Subjective Social Status, and Perceived Stress: Associations with Stress Physiology and Executive Functioning. Behavioral Medicine. 2015;41(3):145-54. doi: 10.1080/08964289.2015.1024604.

56. Roy AL, McCoy DC, Raver CC. Instability versus quality: residential mobility, neighborhood poverty, and children's self-regulation. Dev Psychol. 2014;50(7):1891-6. Epub 2014/05/19. doi: 10.1037/a0036984. PubMed PMID: 24842459.

57. Yeniad N, Malda M, Mesman J, van Ijzendoorn MH, Emmen RAG, Prevoo MJL. Cognitive flexibility children across the transition to school: A longitudinal study. Cognitive Development. 2014;31:35-47. doi: <https://doi.org/10.1016/j.cogdev.2014.02.004>.

58. McDermott JM, Pérez-Edgar K, Fox NA. Variations of the flanker paradigm: assessing selective attention in young children. Behav Res Methods. 2007;39(1):62-70. Epub 2007/06/08. doi: 10.3758/bf03192844. PubMed PMID: 17552472.

59. Micalizzi L, Brick LA, Flom M, Ganiban JM, Saudino KJ. Effects of socioeconomic status and executive function on school readiness across levels of household chaos. Early Child Res Q. 2019;47:331-40. Epub 2019/02/10. doi: 10.1016/j.ecresq.2019.01.007. PubMed PMID: 31341348.

60. Chang SM, Walker SP, Grantham-Macgregor S, Powell CA. Early childhood stunting and later fine motor abilities. Developmental Medicine & Child Neurology. 2010;52(9):831-6. doi: 10.1111/j.1469-8749.2010.03640.x.

61. ZIMSTAT, UNICEF. Zimbabwe Multi-Indicator Cluster Survey 2019; Snapshots of Key Findings. <https://www.unicef.org/zimbabwe/>: 2019.

62. Gochyyev P., Mizunoya S., M. C. Validity and reliability of the MICS educational Module. Data and Analytics Section, Division of Data, Research and Policy, : UNICEF, New York., 2019.

63. Gove A, Wetterberg A. The Early Grade Reading Assessment: Applications and Interventions to Improve Basic Literacy. RTI, 2011.

64. Abubakar A. Use of EGRA to assess inidividual literacy. Personal communication ed2019.

65. Graham J, Kelly S. How Effective Are Early Grade Reading Interventions? A Review of the Evidence. World Bank, 2018.

66. Tofail F, Fernald LCH, Das KK, Rahman M, Ahmed T, Jannat KK, et al. Effect of water quality, sanitation, hand washing, and nutritional interventions on child development in rural Bangladesh (WASH Benefits Bangladesh): a cluster-randomised controlled trial. The Lancet Child & Adolescent Health. 2018;2(4):255-68. doi: 10.1016/S2352-4642(18)30031-2.

67. Stone LL, Janssens JMAM, Vermulst AA, Van Der Maten M, Engels RCME, Otten R. The Strengths and Difficulties Questionnaire: psychometric properties of the parent and teacher version in children aged 4–7. BMC Psychology. 2015;3(1):4. doi: 10.1186/s40359-015-0061-8.

68. Skinner D, Sharp C, Marais L, Serekoane M, Lenka M. Assessing the value of and contextual and cultural acceptability of the Strength and Difficulties Questionnaire (SDQ) in evaluating mental health problems in HIV/AIDS affected children. Int J Ment Health. 2014;43(4):76-89. Epub 2015/04/30. doi: 10.1080/00207411.2015.1009314. PubMed PMID: 27087701.

69. Ocansey ME, Adu-Afarwuah S, Kumordzie SM, Okronipa H, Young RR, Tamakloe SM, et al. Prenatal and postnatal lipid-based nutrient supplementation and cognitive, social-emotional, and motor function in preschool-aged children in Ghana: a follow-up of a randomized controlled trial. Am J Clin Nutr. 2019;109(2):322-34. Epub 2019/02/06. doi: 10.1093/ajcn/nqy303. PubMed PMID: 30721937; PubMed Central PMCID: PMCPMC6367954.

70. Loeb M, Cappa C, Crialesi R, de Palma E, Loeb M, Cappa C, et al. Measuring child functioning: the Unicef/ Washington Group Module. Salud Pública de México. 2017;59(4):485-7. doi: 10.21149/8962.

71. Massey M. The development and testing of a module on child functioning for identifying children with disabilities on surveys. II: Question development and pretesting. Disabil Health J. 2018;11(4):502-9. doi: <https://doi.org/10.1016/j.dhjo.2018.06.006>.

72. Cappa C, Mont D, Loeb M, Misunas C, Madans J, Comic T, et al. The development and testing of a module on child functioning for identifying children with disabilities on surveys. III: Field testing. Disabil Health J. 2018;11(4):510-8. doi: 10.1016/j.dhjo.2018.06.004.

73. Dunne T, Chandna J, Majo F, Chasekwa B, Ntozini R, Prendergast A, et al. G437 Validity of the washington group module on child functioning in 2-year-old children; disability outcome of the shine trial. Archives of disease in childhood. 2020;105(Suppl 1):A158-A. doi: 10.1136/archdischild-2020-rcpch.378.

74. Gladstone M, Lancaster GA, Umar E, Nyirenda M, Kayira E, van den Broek NR, et al. The Malawi Developmental Assessment Tool (MDAT): The Creation, Validation, and Reliability of a Tool to Assess Child Development in Rural African Settings. PLOS Medicine. 2010;7(5):e1000273. doi: 10.1371/journal.pmed.1000273.

75. Norman K, Smoliner C, Kilbert A, Valentini L, Lochs H, Pirlich M. Disease-related malnutrition but not underweight by BMI is reflected by disturbed electric tissue properties in the bioelectrical impedance vector analysis. The British journal of nutrition. 2008;100(3):590-5. Epub 2008/02/01. doi: 10.1017/s0007114508911545. PubMed PMID: 18234142.

76. Wells JCK. Body composition of children with moderate and severe undernutrition and after treatment: a narrative review. BMC Medicine. 2019;17(1):215. doi: 10.1186/s12916-019-1465-8.

77. Wells JC, Hawton K, Darch T, Lunn PG. Body composition by 2H dilution in Gambian infants: comparison with UK infants and evaluation of simple prediction methods. The British journal of nutrition. 2009;102(12):1776-82. Epub 2009/08/18. doi: 10.1017/s0007114509991255. PubMed PMID: 19682404.

78. Wells JC, Devakumar D, Grijalva-Eternod CS, Manandhar DS, Costello A, Osrin D. Blood pressure and the capacity-load model in 8-year-old children from Nepal: Testing the contributions of kidney size and intergenerational effects. American journal of human biology : the official journal of the Human Biology Council. 2016;28(4):555-65. Epub 2016/02/06. doi: 10.1002/ajhb.22829. PubMed PMID: 26848931.

79. Abera M, Tesfaye M, Admassu B, Hanlon C, Ritz C, Wibaek R, et al. Body composition during early infancy and developmental progression from 1 to 5 years of age: the Infant Anthropometry and Body Composition (iABC) cohort study among Ethiopian children. The British journal of nutrition. 2018;119(11):1263-73. Epub 2018/05/18. doi: 10.1017/s000711451800082x. PubMed PMID: 29770755.

80. Wells JCK. The programming effects of early growth. Early Human Development (2007). 2007;83:743-8.

81. Lelijveld N. Long-term effects of severe acute malnutrition on growth, body composition, and function; a prospective cohort study in Malawi 2016.

82. Bartz S, Mody A, Hornik C, Bain J, Muehlbauer M, Kiyimba T, et al. Severe acute malnutrition in childhood: hormonal and metabolic status at presentation, response to treatment, and predictors of mortality. The Journal of clinical endocrinology and metabolism. 2014;99(6):2128-37. Epub 2014/03/13. doi: 10.1210/jc.2013-4018. PubMed PMID: 24606092; PubMed Central PMCID: PMCPMC4037734.

83. Liddle K, O'Callaghan M, Mamun A, Najman J, Williams G. Comparison of body mass index and triceps skinfold at 5 years and young adult body mass index, waist circumference and blood pressure. Journal of Paediatrics and Child Health. 2012;48(5):424-9. doi: <https://doi.org/10.1111/j.1440-1754.2011.02247.x>.

84. Furtado JM, Almeida SM, Mascarenhas P, Ferraz ME, Ferreira JC, Vilanova M, et al. Anthropometric features as predictors of atherogenic dyslipidemia and cardiovascular risk in a large population of school-aged children. PloS one. 2018;13(6):e0197922-e. doi: 10.1371/journal.pone.0197922. PubMed PMID: 29856786.

85. Ivanovic DM. Does undernutrition during infancy inhibit brain growth and subsequent intellectual development? Nutrition. 1996;12(7):568-71. doi: <http://dx.doi.org/10.1016/S0899-9007(97)85097-6>.

86. Lelijveld N, Seal A, Wells JC, Kirkby J, Opondo C, Chimwezi E, et al. Chronic disease outcomes after severe acute malnutrition in Malawian children (ChroSAM): a cohort study. The Lancet Global health. 2016;4(9):e654-62. Epub 2016/07/30. doi: 10.1016/s2214-109x(16)30133-4. PubMed PMID: 27470174; PubMed Central PMCID: PMCPMC4985564.

87. Pomeroy E, Stock JT, Stanojevic S, Miranda JJ, Cole TJ, Wells JC. Trade-offs in relative limb length among Peruvian children: extending the thrifty phenotype hypothesis to limb proportions. PLoS One. 2012;7(12):e51795. Epub 2012/12/29. doi: 10.1371/journal.pone.0051795. PubMed PMID: 23272169; PubMed Central PMCID: PMCPmc3521697.

88. Wells JCK, Devakumar D, Manandhar DS, Saville N, Chaube SS, Costello A, et al. Associations of stunting at 2 years with body composition and blood pressure at 8 years of age: longitudinal cohort analysis from lowland Nepal. European journal of clinical nutrition. 2019;73(2):302-10. Epub 2018/08/30. doi: 10.1038/s41430-018-0291-y. PubMed PMID: 30154534.

89. España-Romero V, Artero EG, Santaliestra-Pasias AM, Gutierrez A, Castillo MJ, Ruiz JR. Hand span influences optimal grip span in boys and girls aged 6 to 12 years. J Hand Surg Am. 2008;33(3):378-84. Epub 2008/03/18. doi: 10.1016/j.jhsa.2007.11.013. PubMed PMID: 18343294.

90. Malina RM, Pena Reyes ME, Tan SK, Little BB. Physical fitness of normal, stunted and overweight children 6-13 years in Oaxaca, Mexico. European journal of clinical nutrition. 2011;65(7):826-34. Epub 2011/03/31. doi: 10.1038/ejcn.2011.44. PubMed PMID: 21448221.

91. Castro-Pinero J, Ortega FB, Artero EG, Girela-Rejon MJ, Mora J, Sjostrom M, et al. Assessing muscular strength in youth: usefulness of standing long jump as a general index of muscular fitness. J Strength Cond Res. 2010;24(7):1810-7. Epub 2010/06/18. doi: 10.1519/JSC.0b013e3181ddb03d. PubMed PMID: 20555277.

92. Armstrong MEG, Lambert MI, Lambert EV. Relationships between different nutritional anthropometric statuses and health-related fitness of South African primary school children. Annals of Human Biology. 2017;44(3):208-13. doi: 10.1080/03014460.2016.1224386.

93. Lang JJ, Tremblay MS, Leger L, Olds T, Tomkinson GR. International variability in 20 m shuttle run performance in children and youth: who are the fittest from a 50-country comparison? A systematic literature review with pooling of aggregate results. British journal of sports medicine. 2018;52(4):276. Epub 2016/09/22. doi: 10.1136/bjsports-2016-096224. PubMed PMID: 27650256.

94. Mayorga-Vega D, Aguilar-Soto P, Viciana J. Criterion-Related Validity of the 20-M Shuttle Run Test for Estimating Cardiorespiratory Fitness: A Meta-Analysis. J Sports Sci Med. 2015;14(3):536-47. PubMed PMID: 26336340.

95. Castro-Piñero J, Artero EG, España-Romero V, Ortega FB, Sjöström M, Suni J, et al. Criterion-related validity of field-based fitness tests in youth: a systematic review. British journal of sports medicine. 2010;44(13):934-43. doi: 10.1136/bjsm.2009.058321.

96. Bustinduy AL, Thomas CL, Fiutem JJ, Parraga IM, Mungai PL, Muchiri EM, et al. Measuring Fitness of Kenyan Children with Polyparasitic Infections Using the 20-Meter Shuttle Run Test as a Morbidity Metric. PLOS Neglected Tropical Diseases. 2011;5(7):e1213. doi: 10.1371/journal.pntd.0001213.

97. Febba A, Sesso R, Barreto GP, Liboni CS, Franco MC, Casarini DE. Stunting growth: association of the blood pressure levels and ACE activity in early childhood. Pediatric nephrology (Berlin, Germany). 2009;24(2):379-86. Epub 2008/09/16. doi: 10.1007/s00467-008-0980-1. PubMed PMID: 18791745.

98. De Lucia Rolfe E, de França GVA, Vianna CA, Gigante DP, Miranda JJ, Yudkin JS, et al. Associations of stunting in early childhood with cardiometabolic risk factors in adulthood. PLOS ONE. 2018;13(4):e0192196. doi: 10.1371/journal.pone.0192196.

99. Kaljee L, Munjile K, Menon A, Tembo S, Li X, Zhang L, et al. The ‘Teachers Diploma Program’ in Zambian Government Schools: A Baseline Qualitative Assessment of Teachers’ and Students’ Strengths and Challenges in the Context of a School-Based Psychosocial Support Program. International Education Studies. 2017;Vol. 10:92-103.

100. Wells JCK, Williams JE, Fewtrell M, Singhal A, Lucas A, Cole TJ. A simplified approach to analysing bio-electrical impedance data in epidemiological surveys. Int J Obes. 2007;31(3):507-14. doi: 10.1038/sj.ijo.0803441.

101. Norman K, Stobäus N, Pirlich M, Bosy-Westphal A. Bioelectrical phase angle and impedance vector analysis – Clinical relevance and applicability of impedance parameters. Clinical Nutrition. 2012;31(6):854-61. doi: <https://doi.org/10.1016/j.clnu.2012.05.008>.

102. España-Romero V, Ortega FB, Vicente-Rodríguez G, Artero EG, Rey JP, Ruiz JR. Elbow position affects handgrip strength in adolescents: validity and reliability of Jamar, DynEx, and TKK dynamometers. J Strength Cond Res. 2010;24(1):272-7. Epub 2009/12/08. doi: 10.1519/JSC.0b013e3181b296a5. PubMed PMID: 19966590.

103. Leger LA, Lambert J. A maximal multistage 20-m shuttle run test to predict VO2 max. European journal of applied physiology and occupational physiology. 1982;49(1):1-12. Epub 1982/01/01. doi: 10.1007/bf00428958. PubMed PMID: 7201922.

104. Brazendale K, Decker L, Hunt ET, Perry MW, Brazendale AB, Weaver RG, et al. Validity and Wearability of Consumer-based Fitness Trackers in Free-living Children. Int J Exerc Sci. 2019;12(5):471-82. PubMed PMID: 30899354.

105. Chasekwa B, Maluccio JA, Ntozini R, Moulton LH, Wu F, Smith LE, et al. Measuring wealth in rural communities: Lessons from the Sanitation, Hygiene, Infant Nutrition Efficacy (SHINE) trial. PLOS ONE. 2018;13(6):e0199393. doi: 10.1371/journal.pone.0199393.

106. Sprunt B, McPake B, Marella M. The UNICEF/Washington Group Child Functioning Module-Accuracy, Inter-Rater Reliability and Cut-Off Level for Disability Disaggregation of Fiji's Education Management Information System. International Journal of Environmental Research and Public Health. 2019;16(5). doi: 10.3390/ijerph16050806.

107. Bhopal S, Roy R, Verma D, Kumar D, Avan B, Khan B, et al. Impact of adversity on early childhood growth & development in rural India: Findings from the early life stress sub-study of the SPRING cluster randomised controlled trial (SPRING-ELS). PLOS ONE. 2019;14(1):e0209122. doi: 10.1371/journal.pone.0209122.

108. Berens AE, Kumar S, Tofail F, Jensen SKG, Alam M, Haque R, et al. Cumulative psychosocial risk and early child development: validation and use of the Childhood Psychosocial Adversity Scale in global health research. Pediatric research. 2019. Epub 2019/05/19. doi: 10.1038/s41390-019-0431-7. PubMed PMID: 31103019; PubMed Central PMCID: PMCPMC6859196.

109. Driscoll KS, Pianta RC. Mothers’ and fathers’ perceptions of conflict and closeness in parent-child relationships during early childhood. Psychology. 2011.

110. Straus MA, Hamby SL, Finkelhor D, Moore DW, Runyan D. Identification of child maltreatment with the Parent-Child Conflict Tactics Scales: development and psychometric data for a national sample of American parents. Child Abuse Negl. 1998;22(4):249-70. Epub 1998/05/20. doi: 10.1016/s0145-2134(97)00174-9. PubMed PMID: 9589178.

111. Chibanda D, Mangezi W, Tshimanga M, Woelk G, Rusakaniko P, Stranix-Chibanda L, et al. Validation of the Edinburgh Postnatal Depression Scale among women in a high HIV prevalence area in urban Zimbabwe. Arch Womens Ment Health. 2010;13(3):201-6. Epub 2009/09/18. doi: 10.1007/s00737-009-0073-6. PubMed PMID: 19760051.

112. Coates J, Swindale A, Bilinsky P. Household Food Insecurity Access Scale (HFIAS) for measurement of food access: indicator guide: version 3. 2007.

113. Gandure S, Drimie S, Faber M. Food Security Indicators after Humanitarian Interventions Including Food Aid in Zimbabwe. Food and nutrition bulletin. 2010;31(4):513-23. doi: 10.1177/156482651003100405.

114. Young SL, Boateng GO, Jamaluddine Z, Miller JD, Frongillo EA, Neilands TB, et al. The Household Water InSecurity Experiences (HWISE) Scale: development and validation of a household water insecurity measure for low-income and middle-income countries. BMJ Global Health. 2019;4(5):e001750. doi: 10.1136/bmjgh-2019-001750.

115. Akoglu H. User's guide to correlation coefficients. Turk J Emerg Med. 2018;18(3):91-3. Epub 20180807. doi: 10.1016/j.tjem.2018.08.001. PubMed PMID: 30191186; PubMed Central PMCID: PMCPMC6107969.

1. Note that the Plus EF tool was only added towards the end of this study and so data for Plus EF are not presented in this paper. [↑](#footnote-ref-2)
