# Supplementary material for: Characterising school-age health and function in rural Zimbabwe using the SAHARAN toolbox
Source: PLoS One. 2023 May 11;18(5):e0285570. doi: 10.1371/journal.pone.0285570 (PMC10174535; doi:10.1371/journal.pone.0285570)
Supplement: Attachment — Submitted filename: Response to reviewers.docx [file pone.0285570.s002.docx]

PONE-D-21-32266

Characterising school-age health and function in rural Zimbabwe using the SAHARAN toolbox

Dear PLOS, Please find our response to the reviewer’s comments below.

Reviewers' comments:

Reviewer's Responses to Questions

**Comments to the Author**

1. Is the manuscript technically sound, and do the data support the conclusions?

The manuscript must describe a technically sound piece of scientific research with data that supports the conclusions. Experiments must have been conducted rigorously, with appropriate controls, replication, and sample sizes. The conclusions must be drawn appropriately based on the data presented.

Reviewer #1: Partly

Reviewer #2: Yes

2. Has the statistical analysis been performed appropriately and rigorously?

Reviewer #1: No

Reviewer #2: Yes

3. Have the authors made all data underlying the findings in their manuscript fully available?

The [PLOS Data policy](http://www.plosone.org/static/policies.action#sharing) requires authors to make all data underlying the findings described in their manuscript fully available without restriction, with rare exception (please refer to the Data Availability Statement in the manuscript PDF file). The data should be provided as part of the manuscript or its supporting information, or deposited to a public repository. For example, in addition to summary statistics, the data points behind means, medians and variance measures should be available. If there are restrictions on publicly sharing data—e.g. participant privacy or use of data from a third party—those must be specified.

Reviewer #1: Yes

Reviewer #2: Yes

4. Is the manuscript presented in an intelligible fashion and written in standard English?

PLOS ONE does not copyedit accepted manuscripts, so the language in submitted articles must be clear, correct, and unambiguous. Any typographical or grammatical errors should be corrected at revision, so please note any specific errors here.

Reviewer #1: Yes

Reviewer #2: Yes

5. Review Comments to the Author

Please use the space provided to explain your answers to the questions above. You may also include additional comments for the author, including concerns about dual publication, research ethics, or publication ethics. (Please upload your review as an attachment if it exceeds 20,000 characters)

Reviewer #1: The manuscript brings relevant data and results; however, the paper has a lot of information, making it difficult for the reader to understand what gaps the study aims at closing.
Writing and format editing is required.
Based on the abstract, it seems that the authors are divided between two approaches: a) to describe the SAHARAN toolbox development, or b) to verify the association between nutritional status, physical and cognitive function. The first major change that needs to be addressed is to adequately define which are the aims of the present study and to focus the description of the entire manuscript accordingly.

**Response: We apologise for the lack of clarity in the manuscript. The reviewer has correctly identified both goals of the paper, and we have clarified the dual aims of this paper in both the Abstract and Introduction.**

**Abstract: “School-age (age 5-14 years) is often described as the ‘missing middle’ of childhood because health, growth and development are poorly characterised, particularly in low- and middle-income countries. There is a shortage of school-age assessment tools combining measurements of growth, health, physical and cognitive function. We developed the School-Age Health, Activity, Resilience, Anthropometry and Neurocognitive (SAHARAN) toolbox to address this gap, then used it to measure growth, cognitive and physical function in a pilot cohort in rural Zimbabwe.”**

**Final paragraph of Introduction: “First, we describe the development of this toolbox. Second, we use this new test battery to evaluate the associations between growth, physical function and cognitive functional domains in a pilot cohort of school-age Zimbabwean children.”**

In my understanding the data presented here is the baseline, is that correct? Please provide further description of the cohort.

**Response:** **We apologise for the confusion: this was not a baseline assessment of a large study; instead, this was a standalone cohort recruited specifically to pilot this new test battery through a single cross-sectional assessment. We have clarified throughout the manuscript that this was a pilot cohort of 80 children, including in the Methods section where we describe enrolment of the cohort. Table 1 provides a detailed description of the cohort.**

Statistical analysis is also a concerning topic. The paper needs a clear outline prior to data analysis. The authors seem to have analyzed all domains available from the toolbox without defining which variable is considered as outcome, exposure, mediator, or adjusting variable.

**Response: We have considerably updated the data analysis section in both the main text and supplementary information. We have improved the clarity of defining the exposures and outcomes before presenting the results.**

As a consequence, Figures 2 and 3 have different outcome variables, making it very confusing for the reader.

**Response: We have updated figure 2 to have a consistent y-axis between graphs, thereby illustrating associations using the same outcome variables in each row, with skinfold thickness, lean mass index and physical function on rows 1, 2 and 3 respectively. We have also added an additional label for each horizontal row for clarity. Similarly in figure 3, we have used consistent y-axes on each row with MPI as the outcome, with the exception of figure 3f (fine motor as the outcome).**

A few descriptive figures/tables, only illustrating the variables, would be interesting.

**Response: Thank you for this suggestion. Additional figures S2, S3 and S4 have been drawn to illustrate the variables, exposures and outcomes being explored. These complement the results tables and data analysis plans.**

Table 1 needs formatting revision to be fit for publication.

**Response:** **This has been corrected in keeping with PLoS guidelines**

Reviewer #2: Reviewer’s comment
Abstract: try to reduce abbreviations in the abstract section

**Response: We have removed all abbreviations except for the standard anthropometry terms, and we believe this has improved readability of the abstract.**

Line 24-27: The introduction needs to re-write clearly and attract the readers.

**Response: This section has been rewritten for clarity**

Line 28-36: The ‘SAHARAN toolbox’ seems more technical and should be explained more for all readers.

**Response: Additional description has been added, as suggested.**

Line 39: The number isn’t written in the figure at the beginning of sentences. Instead, you can write it in full like ‘Eighty’.

**Response: This has been corrected**

Line 124: Do you have justification why you’ve started the methods by Conceptual framework? Could you explain how you’ve used the framework, borrowed or adopted from previous work

**Response: We have added an explanation: the conceptual framework was used to define how the tests were selected and to guide the analysis. In addition, the conceptual framework allowed us to identify the underlying environmental factors. This has been added to the start of the methods section, with reference to Elizabeth Prado’s work mentioned as a guide from previous work.**

Line 334: When you say’ caregiver questionnaire, is that not include parents?

**The caregiver questionnaire was delivered to the primary caregiver, which could be a parent, or any other primary caregiver (eg the grandmother) as detailed in Table 1. This has been clarified in the text line 559.**

6. PLOS authors have the option to publish the peer review history of their article ([what does this mean?](https://journals.plos.org/plosone/s/editorial-and-peer-review-process#loc-peer-review-history)). If published, this will include your full peer review and any attached files.


If you choose “no”, your identity will remain anonymous but your review may still be made public.


**Do you want your identity to be public for this peer review?** For information about this choice, including consent withdrawal, please see our [Privacy Policy](https://www.plos.org/privacy-policy).

Reviewer #1: No

Reviewer #2: No

[NOTE: If reviewer comments were submitted as an attachment file, they will be attached to this email and accessible via the submission site. Please log into your account, locate the manuscript record, and check for the action link "View Attachments". If this link does not appear, there are no attachment files.]

While revising your submission, please upload your figure files to the Preflight Analysis and Conversion Engine (PACE) digital diagnostic tool, <https://pacev2.apexcovantage.com/>. PACE helps ensure that figures meet PLOS requirements. To use PACE, you must first register as a user. Registration is free. Then, login and navigate to the UPLOAD tab, where you will find detailed instructions on how to use the tool. If you encounter any issues or have any questions when using PACE, please email PLOS at [figures@plos.org](mailto:figures@plos.org). Please note that Supporting Information files do not need this step.
